# An integrative taxonomic approach reveals two putatively novel species of phlebotomine sand fly (Diptera: Psychodidae) in Thailand

**DOI:** 10.1186/s13071-024-06640-8

**Published:** 2025-01-06

**Authors:** Samiullah Soomro, Siwaporn Tuangpermsub, Thongchai Ngamprasertwong, Morakot Kaewthamasorn

**Affiliations:** 1 The International Graduate Program of Veterinary Science and Technology (VST), Faculty of Veterinary Science, Chulalongkorn University, Bangkok, 10330 Thailand; 2 Center of Excellence in Veterinary Parasitology, Department of Pathology, Faculty of Veterinary Science, Chulalongkorn University, Bangkok, 10330 Thailand; 3 Veterinary Pathobiology Graduate Program, Faculty of Veterinary Science, Chulalongkorn University, Bangkok, 10330 Thailand; 4 Department of Biology, Faculty of Science, Chulalongkorn University, Bangkok, 10330 Thailand

**Keywords:** Genetic diversity, Phlebotomine, Phylogenetics, Species delimitation, TCS haplotype network, Thailand

## Abstract

**Background:**

The subfamily Phlebotominae comprises 1028 species of sand fly, of which only 90 are recognized as vectors of pathogenic agents such as *Trypanosoma*, *Leishmania*, and *Bartonella*. In Thailand, leishmaniasis—a sand fly-borne disease—is currently endemic, with 36 documented sand fly species. However, many cryptic species likely remain unidentified. To improve our understanding of the distribution, habitat preferences, and role in disease transmission of these sand flies, further research is necessary.

**Methods:**

Sand flies were collected using CDC light traps from 13 locations across four provinces in Thailand between October 2022 and October 2023. Initially, species identification was based on morphological characteristics, employing identification keys, and subsequently confirmed through mitochondrial cytochrome oxidase c subunit I (*COI*) and cytochrome b (*Cytb*) sequencing. Species identities were verified using BLASTN and BOLD searches. Species delimitation was conducted using Automatic Barcode Gap Discovery (ABGD) and Assemble Species by Automatic Partitioning (ASAP) with three substitution models. Additionally, intraspecific and interspecific genetic variation, neutrality tests (including Tajima’s and Fu and Li’s D* tests), phylogenetic analyses, and TCS haplotype network analysis were performed using the obtained sequences.

**Results:**

A total of 3693 phlebotomine sand flies were collected, with 2261 (61.22%) identified as female. Integrative analyses combining morphological data, BLASTN searches, phylogenetic assessments, and species delimitation confirmed the identification of four genera: *Sergentomyia*, *Grassomyia*, *Phlebotomus*, and *Idiophlebotomus*, encompassing 12 species: *Sergentomyia anodontis*, *Se. sylvatica*, *Se. perturbans*, *Se. barraudi*, *Se. hivernus*, *Se. khawi*, *Se. siamensis*, *Grassomyia indica*, *Phlebotomus barguesae*, *Ph. stantoni*, *Idiophlebotomus asperulus*, and *Id. longiforceps*. Furthermore, molecular analysis revealed cryptic and complex species, including two putatively novel species, *Se.* sp. 1 and *Se.* sp. 2, as well as a unique haplotype.

**Conclusions:**

This study, which integrated genetic and morphological identification techniques, identified 12 sand fly species and unveiled cryptic and complex species, including two putatively novel species (*Se.* sp. 1 and *Se.* sp. 2) and a unique haplotype. The findings underscore the utility of mitochondrial genes, combined with species delimitation methodologies, as reliable approaches for identifying diverse sand fly species.

**Graphical Abstract:**

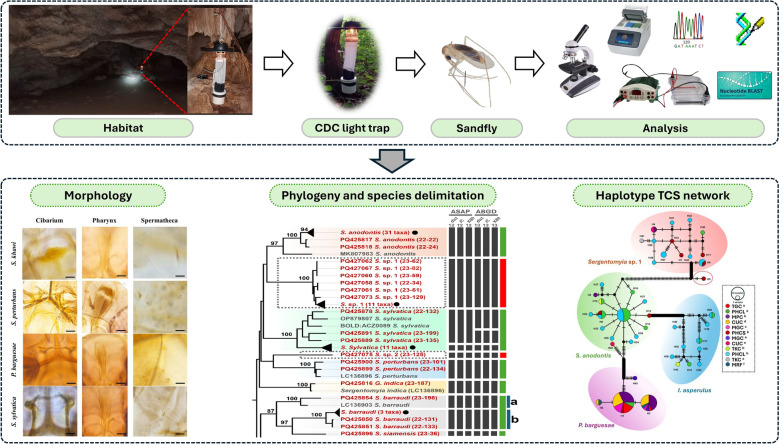

**Supplementary Information:**

The online version contains supplementary material available at 10.1186/s13071-024-06640-8.

## Background

The subfamily Phlebotominae (Diptera: Psychodidae) encompasses 1028 recognized species worldwide, of which only 90 are implicated in the transmission of pathogens such as *Leishmania*, *Trypanosoma*, *Bartonella*, and various viruses [1–5]. A thorough examination of the literature from 1934 to 2019 updated the list to include 34 species of sand flies in Thailand [6], and the discovery of two more species in 2023 [7]—*Phlebotomus shadenae* and *Sergentomyia maiae*—increases the total to 36 species [8]. It is estimated that over one billion people live in regions endemic to leishmaniasis, a sand fly-borne disease, with more than one million new cases of cutaneous leishmaniasis and 30,000 new cases of visceral leishmaniasis reported each year [9]. Once regarded as a rare disease in Thailand, incidences of autochthonous leishmaniasis have increased steadily since 1996, leading to the recognition of Thailand as an endemic region for this disease [10–12]. In Thailand, trypanosomiasis in domestic animals is primarily attributed to *T. lewisi* and *T. evansi* [13, 14]. Notably, Sarataphan et al. [15] reported the infection of a Thai newborn as the first documented case of *T. lewisi*-like illness in humans. Recently, several studies have identified *Trypanosoma* sp. DNA in various species of sand flies [16–18]. To accurately assess the vector potential of sand flies for trypanosomes, however, further investigation is required into the development of this parasite within the insect vector’s gut.

Sand flies and bats often coexist in areas that provide ample opportunities for sand flies to feed on bats or other mammalian hosts [19]. Additionally, many bat species roost in caves, fissures, or the attics of abandoned houses, where the environmental conditions, such as temperature and relative humidity, are believed to be favorable for the breeding and diurnal resting of phlebotomine sand fly species [20]. However, the diversity of sand fly species in Thailand’s caves remains largely unknown owing to the inaccessibility of many caves and the lack of comprehensive studies. Likely, numerous additional species have yet to be recorded [21]. While not all sand fly species act as disease vectors, there is still significant work to be done in terms of conducting thorough assessments of species diversity among cave-dwelling sand flies and accurately identifying the primary natural vectors of trypanosomes [22].

In 2015 alone, more than 25,000 visitors explored caverns in Thailand, highlighting the potential for encounters between tourists and blood-sucking insects, such as sand flies [23]. Identifying sand fly species in these environments has proven challenging owing to their diversity and abundance [24], and many species have been misidentified [25]. Morphological identification, a traditional method of species recognition [26], is particularly difficult for sand flies owing to their small size. This process is further complicated by the labor-intensive nature of specimen preparation, such as mounting and dissection, and the incomplete state of taxonomic knowledge [27]. A vital step in creating effective disease monitoring and management plans is comprehending the variety of neglected tropical disease vectors and enhancing the ability to accurately identify species. DNA barcoding, which uses a relatively short segment of *COI* (mtDNA) for species identification [28], has proven to be an effective tool for identifying sand fly species [21, 29–32]. However, comprehensive *COI* sequence data for sand flies are still lacking from public databases, underscoring the urgent need for additional studies to expand the available genetic information.

Utilizing genetic data for species delimitation offers distinct advantages over traditional morphology-based methods. In recent years, several approaches employing single- or multi-locus datasets have emerged [33, 34]. This study aims to employ conventional morphology-based methods alongside BLASTN and BOLD searches, leveraging single-locus data from the *COI* and *Cytb* genes to identify sand fly species collected from bat caves and other wildlife habitats in Thailand. The taxonomic status of certain species is reevaluated in light of the phylogenetic analyses in this study, revealing two putative novel clades. Accurate species identification of phlebotomine sand flies is crucial for developing effective preventive and control measures, which will, in turn, support the assessment of the risk of local leishmaniasis outbreaks.

## Methods

### Study sites and sample collections

**Fig. 1:**
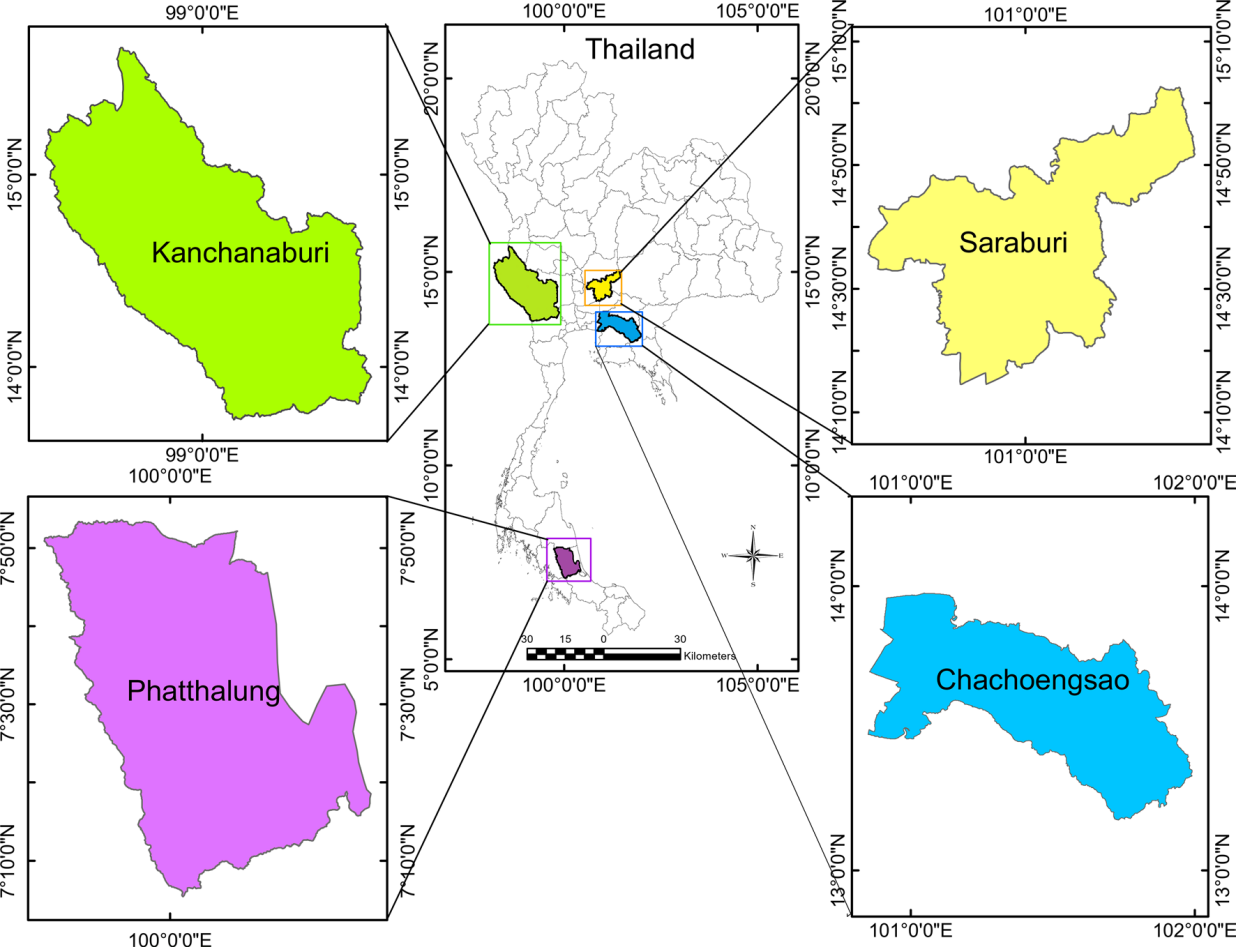
Geographical map illustrates the 13 sampling sites located across four provinces in Thailand where phlebotomine sand fly specimens were collected (see Table 1 for details). The map was generated using ArcGIS version 10.7.1, with the scale bar displayed in kilometers to indicate the distances for each sampling site

This study was undertaken as part of the bat pathogen surveys previously detailed [35–37]. A convenient sampling method was used to collect phlebotomine sand flies from 13 sampling sites in four provinces (Saraburi, Kanchanaburi, Chachoengsao, and Phatthalung) of Thailand from October 2022 to October 2023 (Table 1, Fig. 1). Standard CDC light traps (John W. Hock Company, USA) were set up overnight, approximately 0.5 m above ground, from 6:00 pm to 7:00 am. Traps were installed in underground holes, inside and at the entrance of caves, and in close proximity to wildlife habitats. For specimen processing, insects caught in the nets of CDC traps were transported to the Center of Excellence in Veterinary Parasitology, Chulalongkorn University, and anesthetized in a refrigerator for 5–10 min, as previously reported by Yurkiewicz [38]. An aspirator was used to sort out sand fly specimens from the CDC traps so they could be placed into Petri dishes to examine under a stereomicroscope.

**Table 1:** Number of sand fly specimens collected and their distribution across 13 locations in four provinces of Thailand

Province	Date of collection	Location	Trap no.	Latitude	Longitude	Sample collected					
						Female	Male	Total	No. dissected	No. sequence obtained	
										*COI* (612 bp)	*Cytb* (398–412 bp)
Saraburi	22/10/2022	TGC^a^	Trap 1	14° 31′ 12.6″ N	101° 02′ 13.9″ E	16	5	21	9	9	8
	22/10/2022	CUC^e^	Trap 2	14° 31′ 26.7″ N	101° 01′ 35.9″ E	12	1	13	6	6	6
	08/10/2023	CUC^d^	Trap 3	14° 31′ 27.4″ N	101° 01′ 49″ E	40	7	47	20	20	17
	09/10/2023	CUC^d^	Trap 4	14° 31′ 27.4″ N	101° 01′ 49″ E	2	3	5	2	2	3
Kanchanaburi	28/10/2022	PHCL^a^	Trap 5	14° 24′ 36.6″ N	98° 51′ 13.3″ E	5	1	6	5	5	5
	28/10/2022	PHCL^b^	Trap 6	14° 24′ 37.6″ N	98° 51′ 13.4″ E	14	31	45	7	7	7
	29/10/2022	MGC^a^	Trap 7	14° 21′ 29.8″ N	98° 56′ 11.0″ E	22	9	31	11	11	10
	29/10/2022	MGC^c^	Trap 8	14° 21′ 28.8″ N	98° 56′ 11.4″ E	1950	700	2650	100	15	11
	24/01/2023	MPC^b^	Trap 9	14° 21′ 19.6″ N	98° 56′ 14.1″ E	5	6	11	5	5	5
	25/01/2023	TKC^b^	Trap 10	14° 20′ 34.0″ N	98° 57′ 28.0″ E	22	37	59	11	11	11
	25/01/2023	TKC^a^	Trap 11	14° 20′ 32.5″ N	98° 57′ 28.3″ E	2	4	6	2	2	2
	21/04/2023	MGC^c^	Trap 12	14° 21′ 28.8″ N	98° 56′ 11.4″ E	3	8	11	3	3	3
	22/04/2023	PHCL^b^	Trap 13	14° 24′ 37.6″ N	98° 51′ 13.4″ E	24	39	63	12	11	10
	22/04/2023	PHCS^b^	Trap 14	14° 24′ 35.7″ N	98° 51′ 14.5″ E	28	52	80	14	14	14
	19/06/2023	PHCL^a^	Trap 15	14° 24′ 36.6″ N	98° 51′ 13.3″ E	36	11	47	18	18	18
	19/06/2023	PHCL^b^	Trap 16	14° 24′ 37.6″ N	98° 51′ 13.4″ E	46	19	65	23	23	23
	26/08/2023	MPC^b^	Trap 17	14° 21′ 19.6″ N	98° 56′ 14.1″ E	5	79	84	5	5	5
	27/08/2023	MPC^b^	Trap 18	14° 21′ 19.6″ N	98° 56′ 14.1″ E	16	412	428	10	10	9
Chachoengsao	09/09/2023	MRF^f^	Trap 19	13° 28′ 54″ N	101° 27′ 32″ E	4	1	5	4	4	4
	10/09/2023	MRF^f^	Trap 20	13° 28′ 54″ N	101° 27′ 32″ E	3	3	6	3	3	3
	11/09/2023	MRF^f^	Trap 21	13° 28′ 54″ N	101° 27′ 32″ E	1	1	2	1	1	1
	12/09/2023	MRF^f^	Trap 22	13° 28′ 54″ N	101° 27′ 32″ E	2	1	3	2	2	1
Phatthalung	02/10/2023	PWHR^g^	Trap 23	7° 35′ 08.9″ N	99° 51′ 15″ E	1	0	1	1	1	0
	03/10/2023	PWHR^g^	Trap 24	7° 35′ 08.9″ N	99° 51′ 15″ E	1	1	2	1	0	0
	04/10/2023	PWHR^g^	Trap 25	7° 35′ 08.9″ N	99 °51′ 15″ E	1	1	2	1	1	1
Total						2261	1432	3693	276	189	177

The following letter codes represent the sampling locations used in this study: PHCLa: Phra Cave (large), PHCLb: Phra Cave (large), PHCSb: Phra Cave (small), TKCb: Taklor Cave, TKCa: Taklor Cave, TGCa: Tiger Cave, MGCa: Ma Glue Cave, MGCc: Ma Glue Cave, MPCb: Manow Phee Cave, CUCd: Chulalongkorn University Campus, CUCe: Chulalongkorn University Campus, MRFf: Murrha Farm, PWHRg: Phatthalung Wildlife Husbandry Research Station

The following labels refer to specific sampling areas: ^a^cave entrance, ^b^inside cave, ^c^cave underground, ^d^forest, ^e^banana farm, ^f^buffalo farm, ^g^wildlife sanctuary

### Dissection and morphological identification

For dissection, all female specimens from each trap were processed when fewer than five individuals were collected. When more than 13 specimens were obtained, 50% were dissected. An exception was made for trap no. 8, where only 5.3% (100 specimens) were dissected owing to the large total collection size (1950 individuals). Female sand flies were dissected under a stereomicroscope by removing the head and the posterior abdominal segments containing the spermathecae. These segments were mounted on glass slides using Hoyer’s medium for morphological identification, focusing on key structures such as the cibarium, pharynx, and spermathecae [18, 22, 39]. The thorax, abdomen, and legs were preserved for subsequent molecular analysis.

### Genomic DNA extraction

For genomic DNA (gDNA) extraction, the thorax, abdomen, and legs of 193 dissected female sand flies were processed individually. Each sand fly sample was placed into a 1.5-mL microcentrifuge tube, crushed, and homogenized using pastels in 1× phosphate-buffered saline (PBS). The NucleoSpin^®^ Tissue kit (Macherey–Nagel, Germany) was employed for gDNA extraction following the manufacturer’s protocol, with a minor modification during the elution stage. Specifically, the elution buffer volume was reduced to 25 µl in the first round and 20 µl in the second round. The concentration of the extracted gDNA was measured using a NanoDrop Lite spectrophotometer (Thermo Fisher Scientific, USA), and the samples were stored at −20 °C until further use.

### Polymerase chain reaction (PCR)

PCR was employed to amplify mtDNA *COI* and *Cytb* fragments from the extracted gDNA using two sets of previously published primers [40, 41] (Supplementary Table S1). The primers used for the amplification of the *COI* gene were slightly modified by removing a guanine (G) residue to optimize the melting temperature (*T*_m_). The thermal cycling conditions were regulated according to the protocols specified for each primer set (Supplementary Table S2). PCR reactions were carried out using either an Axygen^®^ MaxyGene II Thermal Cycler (Life Sciences, USA) or a MiniAmp™ Thermal Cycler (Applied Biosystems™, USA). Each reaction had a total volume of 12.5 µl, consisting of 2.75 µl sterile distilled water, 0.375 µl of each forward and reverse primer (final concentration 0.3 μM), 1.75 µl dNTPs, 6.25 µl of 2× KOD Fx Neo Buffer, 0.25 µl KOD Fx Neo polymerase (Toyobo, Japan), and 1 µl of genomic DNA. Sterile distilled water was used as a negative control. PCR products were then loaded with loading dyes into a 1.5% agarose gel stained with ethidium bromide (Bio Basic, USA) and processed in a 0.5× TAE buffer solution using an electrophoresis apparatus (Wealtec, Taiwan) at 100 V, 400 mA, for 40 min. For samples that showed positive results in electrophoresis, the PCR reaction volume was increased from 12.5 µl to 50 µl, and electrophoresis was repeated using the same method. PCR products without nonspecific bands were treated with ExoSAP-IT™ (Applied Biosystems, Lithuania) to remove excess primers and nucleotides. In cases where nonspecific bands were present, the PCR products underwent agarose gel purification. The targeted bands were excised from the gel and purified using the NucleoSpin^®^ Gel and PCR Clean-up kit (Macherey–Nagel, Germany), following the manufacturer’s instructions. The purified PCR products were then sequenced bidirectionally using Sanger sequencing, provided by the commercial sequencing service U2Bio Co., Ltd. (https://www.u2bio.co.th/home).

### Sequence processing, genetic diversity analyses, and BLASTN and BOLD searches

As a quality control measure, the nucleotide sequences and corresponding chromatograms generated in this study were visually inspected, manually edited, and trimmed to remove low-quality regions before generating consensus sequences using BioEdit version 7.2.5 [42]. Primer sequences were excluded from the final consensus sequences, and any sequences exhibiting poor quality or unclear chromatograms were omitted from further analysis. To verify the absence of internal stop codons and ensure accurate reading frames, protein-coding gene alignments were translated into amino acid sequences using the ExPasy database (https://www.expasy.org/). Genetic diversity indices were calculated using DnaSP version 6.12.03 [43], including the average number of nucleotide differences (k), haplotype diversity (Hd), nucleotide diversity (π), number of variable sites (VS), number of haplotypes (H), percentage of G + C content (GC%), along with neutrality tests such as Tajima’s D and Fu and Li’s D*. The trimmed *COI* and *Cytb* sequences were then compared against the GenBank™ database using BLASTN to assess query coverage and percent identity. Additionally, species identification for *COI* sequences was corroborated using the Barcode of Life Database (BOLD) (https://www.boldsystems.org/).

### Phylogenetic analyses, species delimitations, and TCS haplotype networks

A neighbor-joining (NJ) tree was constructed to analyze barcode data, utilizing Kimura’s 2-Parameter (K2P) nucleotide substitution model implemented in MEGA 11 [44]. The analysis followed the default settings, as described in the methodologies of Polseela et al. [21] and Kumar et al. [30]. The tree files were further refined using Figtree software (version 1.4.3) to improve clarity (http://tree.bio.ed.ac.uk/software/figtree/). Reference sequences of phlebotomine sand flies were retrieved from the GenBank database to create phylogenetic trees based on *COI* and *Cytb* genes (Supplementary Table S3). Species delimitation was conducted using the *COI* and *Cytb* sequences, employing two approaches: Automatic Barcode Gap Discovery (ABGD) [45] and Assemble Species by Automatic Partitioning (ASAP) [46]. Both methods were run on webservers with three substitution models, comprising simple-distance (p-distance), JC69 [47], and K2P [48]. For ABGD, the maximum intraspecific distance (Pmax) and minimum intraspecific distance (Pmin) were set to the default values of 0.1 and 0.001, respectively, with a default barcode gap width of 1.5. Recursive partitions were considered with a prior maximal distance of *P* = 5.99 × 10^−2^ after the run. For ASAP, each analysis was performed with ten replicates, and the species partition with the lowest ASAP score was selected as the most accurate to ensure the consistency of the results, following the methodology of Sivayyapram et al. [49]. In addition, TCS haplotype network analysis was performed for both genes (*COI* 613 bp and *Cytb* 398 bp), with the sequences concatenated using MEGA 11 software. The concatenated sequences (1011 bp), as well as the individual sequences of both genes, were used to generate and visualize the TCS haplotype network for the four dominant phlebotomine species identified in this study, utilizing Population Analysis with Reticulate Tree (PopART) software, version 1.7 [50].

## Results

### Sand fly collection

A total of 3693 phlebotomine sand flies were collected from four provinces in Thailand: Saraburi, Kanchanaburi, Chachoengsao, and Phatthalung. Of these, 2261 (61.22%) were female. Notably, 2650 specimens were captured using trap no. 8 from the underground Ma Glue Cave (MGCc), representing the highest proportion (71.76%) of the entire collection. This included 1950 female and 700 male sand flies (52.80% and 18.95% of total catch, respectively). In contrast, the lowest capture rate (0.03%) was recorded for trap no. 23 at the Phatthalung Wildlife Husbandry Research Station (Table 1).

### Dissection and morphological identification

**Fig. 2:**
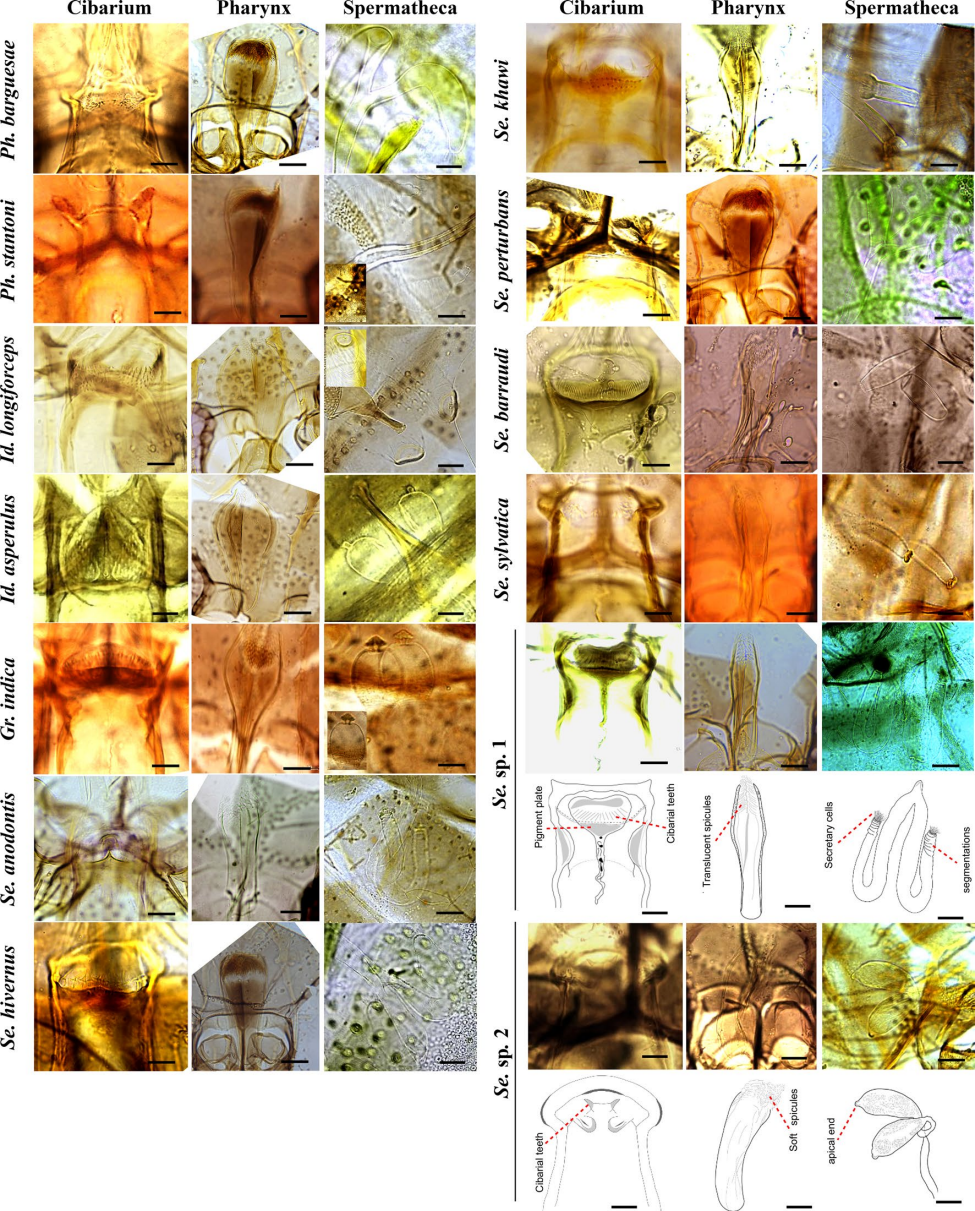
Representative morphological features of phlebotomine sand fly species, highlighting key characteristics of the cibarium, pharynx, and spermathecae, arranged from left to right. The scale bar indicates 20 µm for each feature. For a clearer examination of the morphological details within the micrographs, zooming in is recommended. To facilitate a more detailed visualization of the spermathecae of *Ph. satantoni*, *Id. longiforceps*, and *Gr.indica*, selected features are highlighted within inset illustrations. Additionally, detailed drawings of the cibarium, pharynx, and spermathecae for *Se.* sp. 1 and *Se.* sp. 2 are presented to further aid in morphological comparisons

We dissected 278 female sand fly specimens collected from 25 traps. While all dissected samples from each trap were subjected to PCR and sequencing, an exception was made for trap no. 8, from which only 15 of the 100 specimens were sequenced owing to the large collection size. A total of 189 *COI* sequences and 177 *Cytb* sequences were successfully obtained, although 4 *COI* and 16 *Cytb* PCR products failed to sequence (Table 1). Morphological identification of the corresponding 189 sand fly specimens focused on the cibarium, pharynx, and spermathecae, as illustrated in Fig. 2. This analysis revealed 11 morphospecies in addition to two novel species across four genera, including *Sergentomyia* (7 species), *Grassomyia* (1 species), *Phlebotomus* (2 species), and *Idiophlebotomus* (2 species), with *Sergentomyia* emerging as the dominant genus. The identified species included *Sergentomyia anodontis*, *Se. sylvatica*, *Se. perturbans*, *Se. barraudi*, *Se. hivernus*, *Se. khawi*, *Grassomyia indica*, *Phlebotomus barguesae*, *Ph. stantoni*, *Idiophlebotomus asperulus*, and *Id. longiforceps,* alongside two putative novel species (*Sergentomyia* sp. 1 and *Se.* sp. 2). Our examination of *Se.* sp. 1 revealed that the ventral plate of the cibarium contains 15–16 cibarial teeth (hind teeth), while the dorsal plate exhibits a funnel-shaped patch of dark golden-brown pigment. The spermatheca is tubular in structure, segmented into 8–10 parts, and features secretory cells at its distal end. The spermathecal duct is elongated and broadens toward its terminus. The pharynx is slender with a relatively wide base and is characterized by a robust array of pointed, translucent teeth aligned along the central axis in its posterior quarter. In contrast, *Se.* sp. 2 is characterized by a ventral plate of the cibarium with two prominent cibarial teeth (hind teeth) and the absence of a pigment patch. The spermatheca consists of oblong, oval, or pear-shaped capsules, each with a slightly tapered and rounded apex, forming a rounded apical end. The length of the spermathecal duct remains indeterminate as it was not visible in the specimens examined. The pharynx is slender with a moderately wide base and features a small cluster of fine spicules located in its posterior section. The distinct diagnostic morphological characteristics of these two putative novel species, as illustrated in Fig. 2, confirm that they cannot be assigned to any previously described taxa.

### Cytb sequences and phylogenetic analysis

**Fig. 3:**
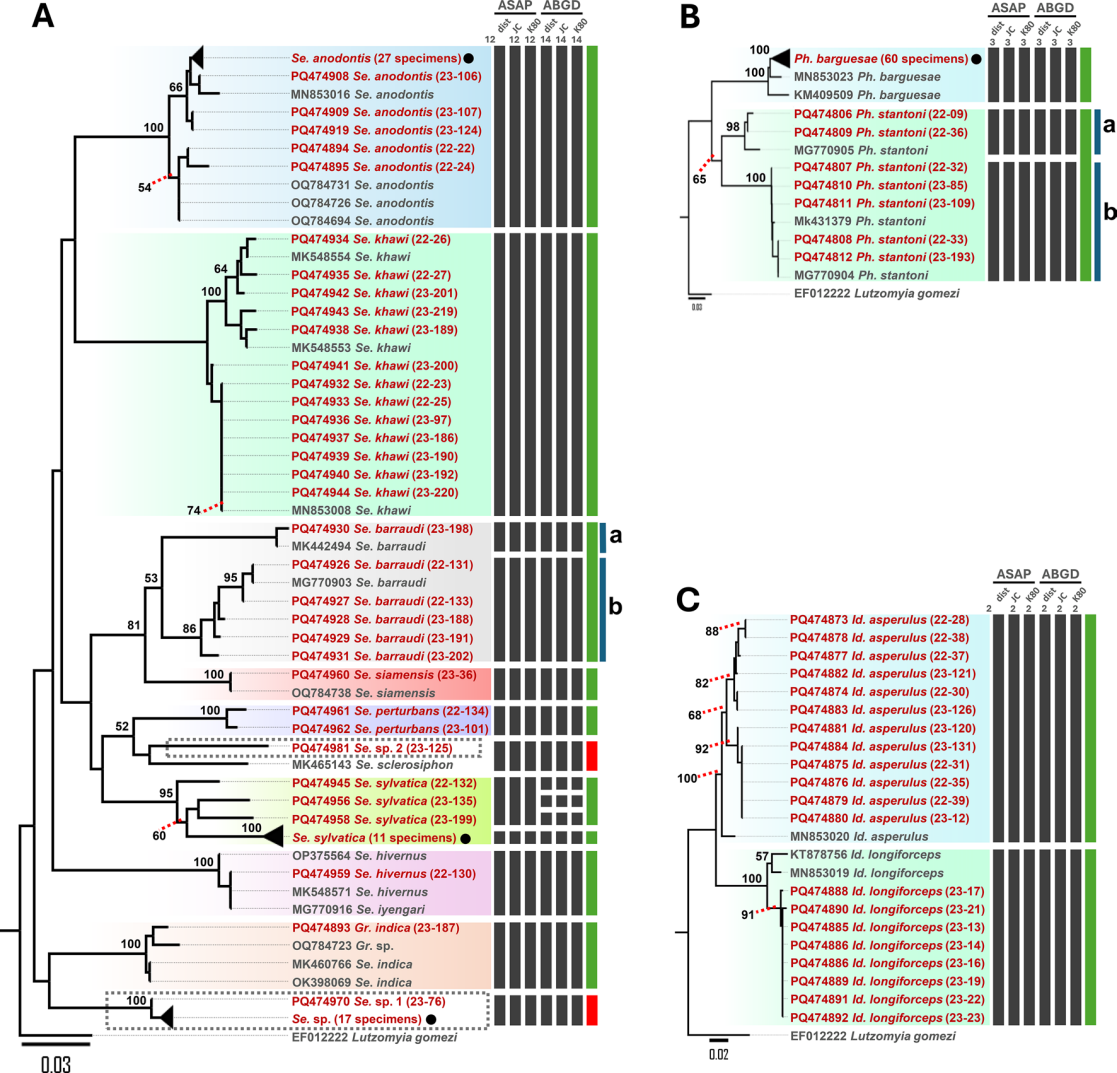
Neighbor-Joining (N-J) tree illustrating the phylogenetic relationships among species within the genera *Sergentomyia* and *Grassomyia* (**A**), *Phlebotomus* (**B**), and *Idiophlebotomus* (**C**), based on mitochondrial *Cytb* sequences analyzed using the Kimura 2-Parameter method. Bootstrap values, derived from 1000 replicates, are displayed above the branches, while values below 50% are omitted. The scale bar indicates divergence percentages: 0.03% for panels **A** and **B**, and 0.02% for **C**. Sequences generated in this study are highlighted in red, whereas reference sequences are depicted in black. Collapsed sequences are represented by black circles (●). Two novel clades are highlighted within dashed rectangles. Additionally, species delimitation based on *Cytb* gene sequences (224 bp) was performed using the ASAP and ABGD web servers, incorporating three substitution models: simple distance (p-distance), Jukes–Cantor (JC69), and Kimura 2-Parameter (K2P). Black bars denote species delineated by each substitution model, green bars represent nominal species, and red bars indicate putative novel species. The first two digits in the parenthesis indicate the year of collection, while digits after hyphen show the voucher ID of specimen. *Lutzomyia gomezi* (EF012222) was utilized as the outgroup

A total of 177 cytochrome b (*Cytb*) sequences, each 398 base pairs in length, were successfully obtained (Table 2). To further validate species identification, BLASTN analysis was conducted for all *Cytb* sequences, with details such as accession numbers, percentage identity, and query coverage provided in Supplementary Table S4. Owing to the limited availability of longer reference sequences in the NCBI GenBank database, a 224 base pair segment was selected for phylogenetic analysis and species delimitation. A total of 203 *Cytb* sequences were included in constructing the neighbor-joining (NJ) phylogenetic tree (Fig. 3A–C). This dataset comprised 177 sequences submitted to NCBI GenBank (accession numbers in Supplementary Table S5) and 25 *Cytb* reference sequences from GenBank, with *Lutzomyia gomezi* (EF012222) serving as an outgroup. The NJ tree clustered the sequences according to their nominal species, with strong bootstrap support (≥ 95%). This pattern was consistent for species with conspecific sequences in GenBank, except for the *Sergentomyia barraudi* clade, which split into subclades a and b, displaying bootstrap values of 86% and 100%, respectively (Fig. 3A). Two sequences (THSF22-134 and THSF23-101) were excluded from the reference sequence analysis owing to the low percentage of identity (less than 91%) in the NCBI GenBank database. However, these sequences were designated as *Se. perturbans* on the basis of morphological identification and a bootstrap value of 100% in the phylogenetic tree. Notably, two putative novel species, *Se.* sp. 1 and *Se.* sp. 2, were identified within the genus *Sergentomyia*. *Se.* sp. 2 consisted of a single sequence (THSF23-125) with a low percentage identity of 90.07% to *Se. sclerosiphon* in the BLASTN results and a low bootstrap value (≤ 48%) in the phylogenetic analysis. Similarly, *Se.* sp. 1 included 18 collapsed sequences that showed low identity (≤ 89%) to *Se. boironis* in BLASTN results but formed a distinct clade with strong bootstrap support (100%) in the phylogeny (Fig. 3A). Within the genus *Phlebotomus*, *Ph. stantoni* was divided into subclades a and b, with bootstrap values of 98% and 100%, respectively (Fig. 3B). Sequences from the genus *Idiophlebotomus* were classified into two distinct species, each with a bootstrap value of 100% (Fig. 3C).

**Table 2:** Distribution and percentage of sand flies across 13 locations in four provinces, as determined by cytochrome c oxidase subunit I (*COI*) and cytochrome b (*Cytb*) gene sequences

Gene	Species	PHCL^a^	PHCL^b^	PHCS^b^	TKC^b^	TKC^a^	MGC^a^	MGC^c^	MPC^b^	TGC^a^	CUC^d^	CUC^e^	MRF^f^	PWHR^g^	Total	Total %
*COI*	*Se. anodontis*	12	14	3	1	0	0	0	1	0	2	0	0	0	33	17.5
	*Se. sylvatica*	2	6	4	0	0	0	1	0	0	1	0	0	0	14	7.4
	*Se. perturbans*	0	0	0	0	0	0	2	0	0	0	0	0	0	2	1.1
	*Se. barraudi*	0	0	0	0	1	0	2	0	0	1	0	2	1	7	3.7
	*Se. hivernus*	0	0	0	0	0	0	1	0	0	0	0	0	0	1	0.5
	*Se. khawi*	1	0	0	0	0	0	0	0	0	2	6	4	1	14	7.4
	*Se*. sp. 1	1	9	5	0	1	0	0	1	0	0	0	0	0	17	9
	*Se*. sp. 2	0	1	0	0	0	0	0	0	0	0	0	0	0	1	0.5
	*Gr. indica*	0	0	0	0	0	0	0	0	0	0	0	1	0	1	0.5
	*Id. asperulus*	7	4	0	1	0	0	0	0	0	0	0	0	0	12	6.3
	*Id. longiforceps*	0	0	0	8	0	0	0	0	0	0	0	0	0	8	4.2
	*Ph. stantoni*	2	2	1	0	0	0	0	0	1	0	0	1	0	7	3.7
	*Ph. barguesae*	1	1	1	1	0	11	13	18	8	16	0	2	0	72	38.1
	Total	26	37	14	11	2	11	19	20	9	22	6	10	2	189	100
*Cytb*	*Se. anodontis*	12	14	2	1	0	0	0	1	0	0	2	0	0	32	18.1
	*Se. sylvatica*	2	6	4	0	0	0	1	0	0	1	0	0	0	14	7.9
	*Se. perturbans*	0	0	0	0	0	0	2	0	0	0	0	0	0	2	1.1
	*Se. barraudi*	0	0	0	0	0	0	2	0	0	1	0	2	1	6	3.4
	*Se. hivernus*	0	0	0	0	0	0	1	0	0	0	0	0	0	1	0.6
	*Se. khawi*	1	0	0	0	0	0	0	0	0	2	6	4	0	13	7.3
	*Se. siamensis*	0	0	0	0	1	0	0	0	0	0	0	0	0	1	0.6
	*Se*. sp. 1	1	9	6	0	1	0	0	1	0	0	0	0	0	18	10.2
	*Se.* sp. 2	0	1	0	0	0	0	0	0	0	0	0	0	0	1	0.6
	*Gr. indica*	0	0	0	0	0	0	0	0	0	0	0	1	0	1	0.6
	*Id. asperulus*	7	4	0	1	0	0	0	0	0	0	0	0	0	12	6.8
	*Id. longiforceps*	0	0	0	8	0	0	0	0	0	0	0	0	0	8	4.5
	*Ph. stantoni*	2	2	1	0	0	1	0	0	1	0	0	1	0	8	4.5
	*Ph. barguesae*	1	0	1	1	0	9	9	17	7	13	0	2	0	60	33.9
	Total	26	36	14	11	2	10	15	19	8	17	8	10	1	177	100.0

The following letter codes represent the sampling locations used in this study: PHCLa: Phra Cave (large), PHCLb: Phra Cave (large), PHCSb: Phra Cave (small), TKCb: Taklor Cave, TKCa: Taklor Cave, TGCa: Tiger Cave, MGCa: Ma Glue Cave, MGCc: Ma Glue Cave, MPCb: Manow Phee Cave, CUCd: Chulalongkorn University Campus, CUCe: Chulalongkorn University Campus, MRFf: Murrha Farm, PWHRg: Phatthalung Wildlife Husbandry Research Station

The following labels refer to specific sampling areas: ^a^cave entrance, ^b^inside cave, ^c^cave underground, ^d^forest, ^e^banana farm, ^f^buffalo farm, ^g^wildlife sanctuary

### Species delimitation using Cytb sequences

Species delimitation was performed using the Assemble Species by Automatic Partitioning (ASAP) and Automatic Barcode Gap Discovery (ABGD) algorithms, which categorized the sequences into 17 and 19 hypothetical species, respectively (Fig. 3A–C). The analysis of species delimitation generally corroborated the phylogenetic tree constructed using the *Cytb* gene, except for the *Se. sylvatica* clade. Notably, all three substitution models employed by both methods suggested the division of *Se. sylvatica* into two and four hypothetical species, respectively. However, morphological and phylogenetic analyses supported the classification of *Se. sylvatica* as a single species, exhibiting a high bootstrap value of 95%. Furthermore, phylogenetic analysis and species delimitation methods indicated that *Se. siamensis* (THSF23-36) is a distinct species separate from the *Se. barraudi* clade (Fig. 3A). Unfortunately, morphological identification was inconclusive in differentiating *Se. siamensis* from the *Se. barraudi* clade. Overall, the integrative results from the morphological assessment, BLASTN analysis, phylogenetic analysis, and species delimitation confirmed the identification of four genera (*Sergentomyia*, *Grassomyia*, *Phlebotomus*, and *Idiophlebotomus*), comprising 12 species including *Sergentomyia anodontis*, *Se. sylvatica*, *Se. perturbans*, *Se. barraudi*, *Se. hivernus*, *Se. khawi*, *Se. siamensis*, *Grassomyia indica*, *Phlebotomus barguesae*, *Ph. stantoni*, *Idiophlebotomus asperulus*, and *Id. longiforceps*, in addition to two putative novel species (*Sergentomyia* sp. 1 and *Sergentomyia* sp. 2) (Fig. 3A–C).

### COI sequences and phylogenetic analysis

**Fig. 4:**
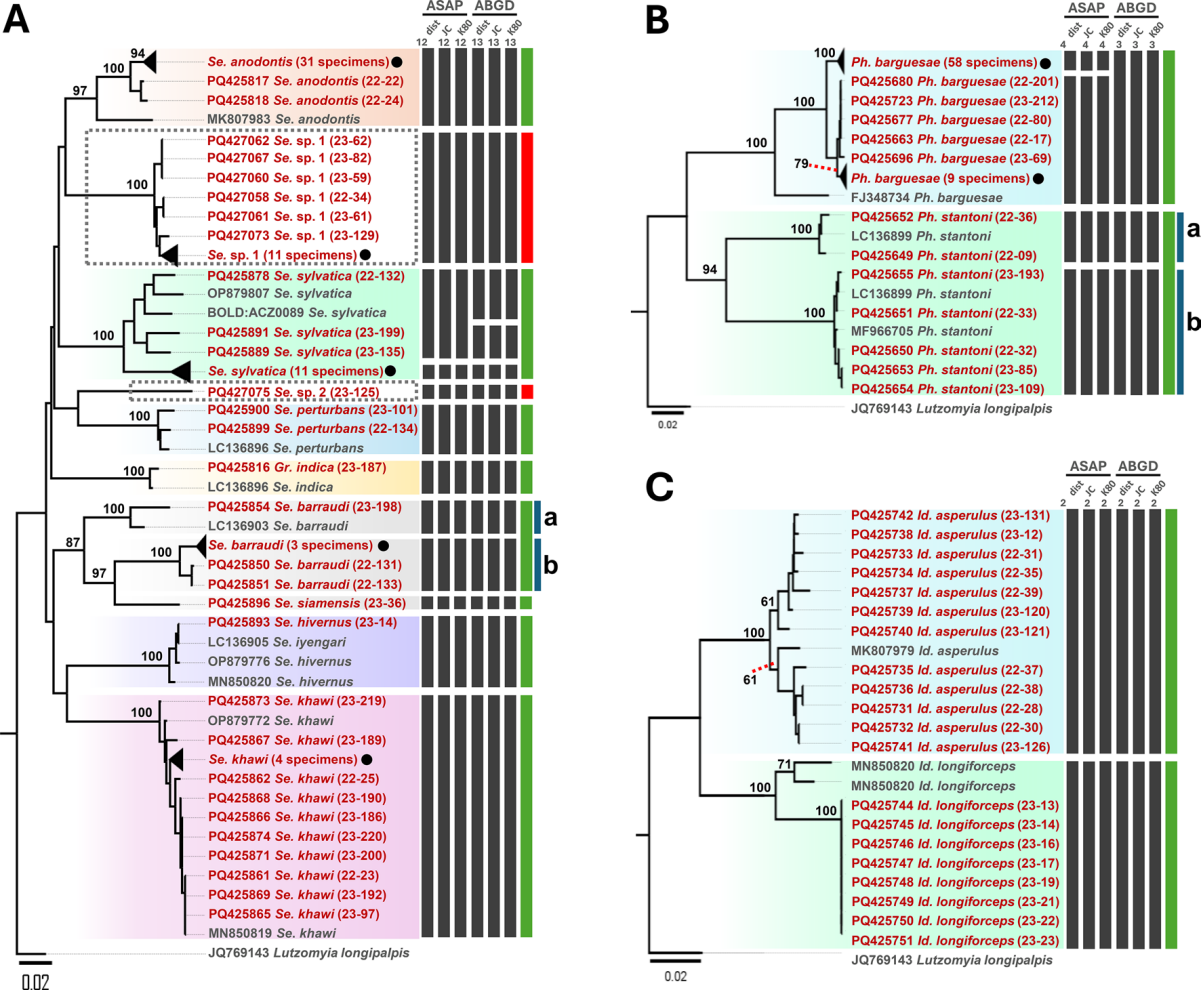
Neighbor-joining (N-J) tree illustrating the phylogenetic relationships among species within the genera *Sergentomyia* and *Grassomyia* (**A**), *Phlebotomus* (**B**), and *Idiophlebotomus* (**C**) based on mitochondrial *COI* sequences (537 bp) using the Kimura 2-Parameter method. Bootstrap values, based on 1000 replicates, are shown above the branches. The bar scale represents a 0.02% divergence. Bootstrap values below 50% are excluded. Sequences obtained in this study are displayed in red, and reference sequences are in black. Collapsed sequences are indicated by black circles (●). Two novel clades are highlighted within dashed rectangles. Species delimitation based on *COI* gene sequences (537 bp) was conducted using ASAP and ABGD webservers with three substitution models: simple distance (p-distance), Jukes-Cantor (JC69), and Kimura 2-Parameter (K2P). Black bars indicate species delineated by each substitution model, green bars denote nominal species, and red bars indicate putative novel species. The first two digits in the parenthesis indicate the year of collection, while digits after hyphen show the voucher ID of specimen. *Lutzomyia longipalpis* (JQ769143) was used as the outgroup

A total of 189 cytochrome c oxidase subunit I (*COI*) sequences were obtained, each comprising 537 base pairs (bp) (Table 2). Subsequent BLASTN and BOLD analyses were performed to further confirm species identification, with the results—including accession numbers, percentage identity, and query coverage—presented in Supplementary Table S4. A comprehensive dataset of 208 *COI* sequences was utilized to construct the neighbor-joining (NJ) phylogenetic tree (Fig. 4A–C), which included the 189 sequences submitted to the NCBI GenBank database (accession numbers provided in Supplementary Table S5) and 18 reference sequences from the GenBank database (Supplementary Table S4), in addition to *Lutzomyia longipalpis* (JQ769143), employed as an outgroup. The NJ tree clustered the sequences according to the sampled nominal species, exhibiting high bootstrap support values (≥ 97%). This clustering pattern was consistently observed for species with conspecific sequences from GenBank, except for the *Sergentomyia barraudi* clade, which was divided into subclades A and B, displaying bootstrap values of 97% and 100%, respectively (Fig. 4A), contradictory to the results obtained from the *Cytb* analysis (Fig. 3A). Similar to the findings from the *Cytb* analysis, two putative novel species within the genus *Sergentomyia* were identified: *Sergentomyia* sp. 1 and *Se.* sp. 2. *Se.* sp. 2 consisted of a single sequence (THSF23-125) that exhibited a low percentage identity of 88.59% with *Se. perturbans* in the BLASTN results. In contrast, *Se.* sp. 1 comprised 17 sequences (collapsed) that also showed a low percentage identity (≤ 90%) with *Se. anodontis* in the BLAST results, while maintaining a high bootstrap value of 100% in the phylogenetic analysis, thus forming a distinct clade (Fig. 4A). Additionally, *Ph. stantoni* in the genus *Phlebotomus* was similarly divided into subclades A and B, both exhibiting a bootstrap value of 100% (Fig. 4B). The sequences associated with the genus *Idiophlebotomus* were classified into two species (Fig. 4C), each displaying bootstrap values of 100%.

### Species delimitation of *COI* sequences

Species delimitation was conducted using the Assemble Species by Automatic Partitioning (ASAP) and Automatic Barcode Gap Discovery (ABGD) algorithms, which classified the sequences into 18 hypothetical species (Fig. 4A–C). The results of the species delimitation analyses generally corroborated the phylogenetic tree constructed using the *Cytb* gene, with notable exceptions observed in the *Sergentomyia sylvatica* (Fig. 4A) and *Phlebotomus barguesae* (Fig. 4B) clades. All three substitution models employed by the ASAP and ABGD methods consistently subdivided the *Se. sylvatica* species into two to three hypothetical species. However, both morphological and phylogenetic analyses supported the classification of the *Se. sylvatica* clade as a single species, as evidenced by a bootstrap value of 95%, consistent with the findings from the *COI* gene analysis. Similarly, the species delimitation methods recognized *Se. siamensis* (THSF23-36) as a distinct species, separate from the *Se. barraudi* clade (Fig. 3A). In contrast, BLASTN and BOLD analyses of the *COI* sequences indicated a high percentage identity (≥ 99.3%) with *Se. barraudi*, while *Cytb* results revealed a high percentage identity (≥ 99.2%) with *Se. siamensis*. In summary, the integrative results from morphological assessments, BLASTN analyses, phylogenetic evaluations, and species delimitation analyses confirmed the presence of four genera (*Sergentomyia*, *Grassomyia*, *Phlebotomus*, and *Idiophlebotomus*), encompassing a total of 11 morphospecies: *Se. anodontis*, *Se. sylvatica*, *Se. perturbans*, *Se. barraudi*, *Se. hivernus*, *Se. khawi*, *Grassomyia indica*, *Ph. barguesae*, *Ph. stantoni*, *Idiophlebotomus asperulus*, and *Id. longiforceps*. Additionally, two putative novel species, *Sergentomyia* sp. 1 and *Se.* sp. 2, were identified (Figs. 3A–C; 4–C).

### Haplotype analysis

The analysis of haplotypes carried out with combined *COI* and *Cytb* sequences from 12 phlebotomine species, along with two putative novel species, revealed a total of 90 haplotypes, as outlined in Table 3. In the case of *Idiophlebotomus asperulus*, haplotypes H4 (*n* = 2) and H5 (*n* = 2) were the most frequently observed, with H8 to H13 representing the least common types. For *Id. longiforceps,* haplotypes H21 (*n* = 3) and H22 (*n* = 2) were the most prevalent, with H23 to H25 representing the least common types. For *Phlebotomus barguesae*, haplotypes H1 (*n* = 27) and H2 (*n* = 16) were the most prevalent, while H3 and H14 were among the least common haplotypes. For *Ph. stantoni*, haplotype H19 (*n* = 2) was the most prevalent, while H15 to H18, and H20 were among the least common haplotypes. For *Sergentomyia anodontis*, haplotype H11 (*n* = 11) was the most common, followed by H3 (*n* = 4), while the least common haplotypes included H1, H9, H10, H12, H13, H15 to H21, and H28. For *Se. barraudi*, each haplotype from H31 to H36 revealed *n* = 1. For *Se. khawi*, haplotypes H37 (*n* = 3) and H41 (*n* = 2) were the most common, with H38 to H40 and H42 to H46 representing the least common types. For *Se. sylvatica,* each haplotype from H47 to H60 revealed *n* = 1. For *Se. hivernus*, *Se. siamensis*, and *Se.* sp. 2, each species revealed a haplotype with *n* = 1. For *Se. perturbans*, each haplotype from H63 and H64 revealed *n* = 1. Among the putative novel species *Se.* sp. 1, haplotype H5 (*n* = 3) was the most prevalent, followed by H25 (*n* = 2); the least common haplotypes included H2, H4, H6 to H8, H14, H22 to H24, H26, H27, and H29.

**Table 3:** Haplotype distribution of sand fly species based on concatenated *COI* (613 bp) and *Cytb* (397–413 bp) sequences collected from 13 locations

Species	Location	No. of sand flies (*n*)	Haplotypes
*Id. asperulus*	TKC^b^	1	H13(1)
	PHCL^a^	3	H4(1), H5(1), H6(1)
	PHCL^b^	8	H4(1), H5(1), H7(1), H8(1), H9(1), H10(1), H11(1), H12(1)
*Id. longiforceps*	TKC^b^	8	H21(3), H22(2), H23(1), H24(1), H25(1)
*Ph. barguesae*	MPC^b^	16	H1(9), H2(7)
	PHCL^a^	1	H1(1)
	TGC^a^	6	H1(5), H2(1)
	PHCS^b^	1	H1(1)
	MGC^c^	5	H1(4), H2(1)
	CUC^d^	13	H1(7), H2(5), H3(1)
	MGC^a^	2	H2(1), H14(1)
	MRF^f^	2	H2(1), H3(1)
*Ph. stantoni*	TGC^a^	1	H15(1)
	PHCL^a^	2	H16(1), H19(1)
	PHCL^b^	2	H17(1), H18(1)
	PHCS^b^	1	H19(1)
	MRF^f^	1	H20(1)
*Gr. indica*	MRF^f^	1	H30(1)
*Se. anodontis*	TKC^b^	1	H12(1)
	PHCL^a^	11	H3(2), H10(1), H11(6), H12(1), H13(1)
	PHCL^b^	13	H3(1), H11(5), H15(1), H16(1), H17(1), H18(1), H19(1), H20(1), H21(1)
	CUC^e^	1	H28(1)
	MPC^b^	1	H1(1)
	PHCS^b^	2	H3(1), H9(1)
*Se. barraudi*	MGC^c^	2	H31(1), H32(1)
	MRF^f^	2	H33(1), H34(1)
	PWHR^g^	1	H35(1)
	CUC^d^	1	H36(1)
*Se. khawi*	CUC^e^	6	H37(1), H38(1), H39(1), H40(1), H41(1), H46(1)
	PHCL^a^	1	H37(1)
	MRF^f^	4	H37(1), H41(1), H42(1), H43(1)
	CUC^d^	2	H44(1), H45(1)
*Se. sylvatica*	MGC^c^	1	H47(1)
	PHCL^b^	6	H48(1), H49(1), H50(1), H57(1), H58(1), H59(1)
	PHCS^b^	4	H51(1), H52(1), H53(1), H54(1)
	PHCL^a^	2	H55(1), H56(1)
	CUC^d^	1	H60(1)
*Se. hivernus*	MGC^u^	1	H61(1)
*Se. siamensis*	TKC^a^	1	H62(1)
*Se. perturbans*	MGC^u^	1	H63(1)
	PHCL^a^	1	H64(1)
*Se.* sp. 1	MPC^b^	1	H2(1)
	PHCL^a^	1	H14(1)
	TKC^a^	1	H29(1)
	PHCS^b^	5	H4(1), H5(1), H6(1), H7(1), H8(1)
	PHCL^b^	9	H5(2), H22(1), H23(1), H24(1), H25(2), H26(1), H27(1)
*Se.* sp. 2	PHCL^b^	1	H65(1)

The numbers in parentheses represent the relative frequency of each haplotype

### TCS haplotype network analysis

**Fig. 5:**
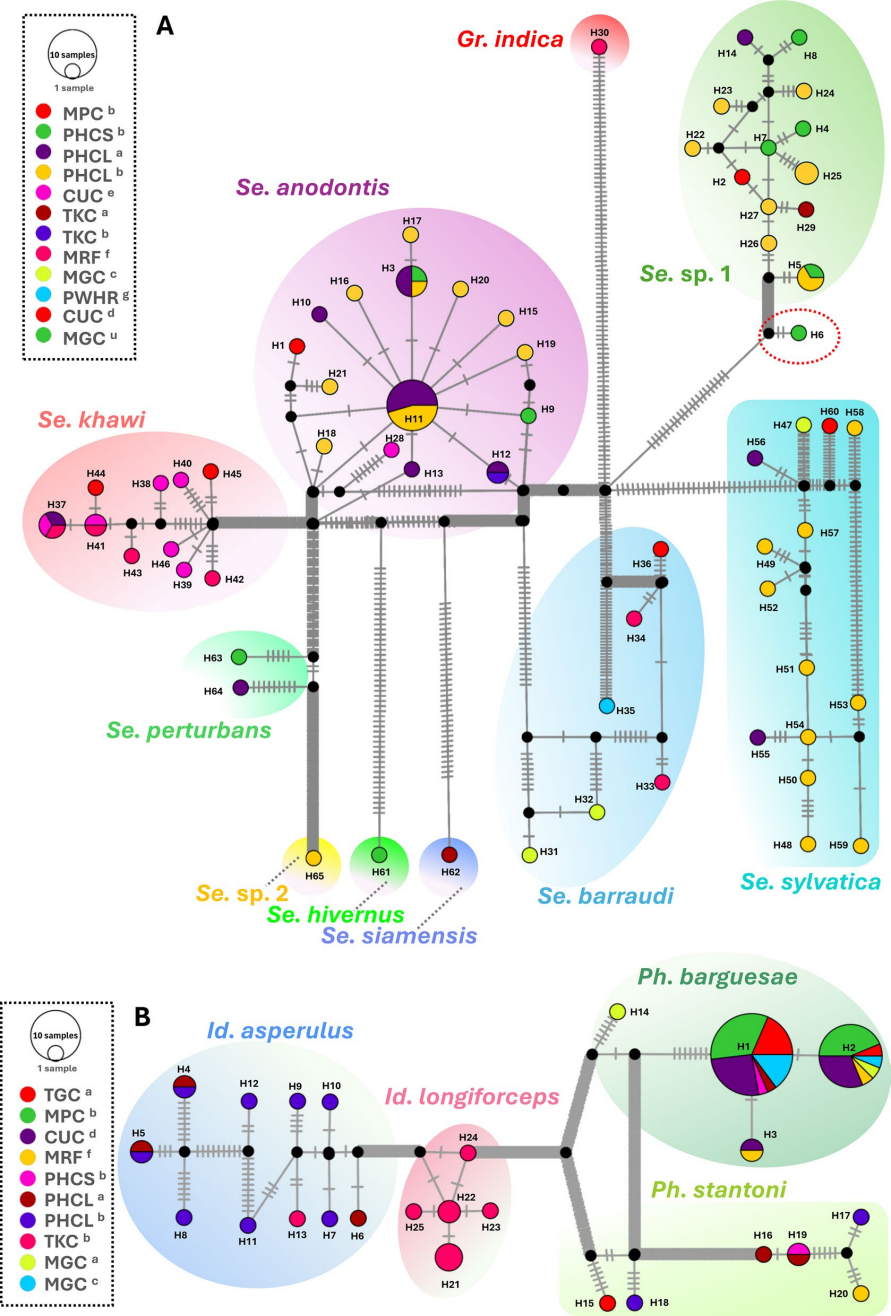
The TCS haplotype network of the four genera *Sergentomyia* and *Grassomyia* (**A**), *Phlebotomus* and *Idiophlebotomus* (**B**), using 158 concatenated *COI* (613 bp) and *Cytb* (397–413 bp) sequences. Each haplotype is depicted as a circle, with the size of the circle proportional to the number of individuals sharing that haplotype. Lines connecting the haplotypes represent nucleotide differences, indicating mutations from a common ancestral haplotype. Missing haplotypes are represented by black dots. The colors corresponding to the sampling locations across provinces are annotated in the accompanying legend, and the letter codes are provided in Tables 1–3

The TCS haplotype network was constructed for concatenated *COI* and *Cytb* sequences (lengths ranging from 1010 to 1026 bp). TCS haplotype network for genus *Sergentomyia* and *Grassomyia* revealed a total of 65 haplotypes categorized into 8 distinct species clusters, including *Sergentomyia anodontis* (15 haplotypes), *Se. khawi* (10 haplotypes), *Se. sylvatica* (14 haplotypes), *Se. barraudi* (6 haplotypes), *Se. perturbans* (2 haplotypes), *Se. hivernus* (1 haplotypes), *Se. siamensis* (1 haplotypes), *Grassomyia indica* (1 haplotypes), and two putative novel spp. *Sergentomyia* sp. 1 (13 haplotypes), and *Se.* sp. 2 (1 haplotypes). While one haplotype (H6) belonging to *Se.* sp. 1 created a separate cluster (Fig. 5A). For *Se. anodontis*, *PHCL*^*b*^ exhibited the highest diversity, containing nine haplotypes (H3, H11, H15, H16, H17, H18, H19, H20, and H21), followed by *PHCL*^*a*^ with 5 haplotypes (H3, H10—H13); the clusters *TKC*^*b*^, *CUC*^*e*^, *MPC*^*b*^, and *PHCS*^*b*^ each had two or fewer haplotypes. For *Se. khawi*, *CUC*^*e*^ exhibited the highest diversity, containing six haplotypes (H37, H38, H39, H40, H41, and H46), followed by *MRF*^*f*^ containing 4 haplotypes (H3, H41, H42, and H43) and thereafter *CUC*^*d*^ and *PHCL*^*a*^ exhibiting 2 (H44 and h45) and 1 haplotype (H37) respectively. For *Se. sylvatica*, *PHCL*^*b*^ exhibited the highest diversity, containing ten haplotypes (H48, H49, H50, H51, H52, H53, H54, H57, H58, and H59) followed by *MGC*^*c*^ and *CUC*^*d*^ each exhibiting 1 haplotype, H47 and H60, respectively. For *Se. barraudi*, *MGC*^*c*^* and MRF*^*f*^ each exhibited 2 haplotypes (H31–H32 and H33–H34), followed by *PWHR*^*g*^ and *CUC*^*d*^ each exhibiting 1 haplotype (H35 and H36, respectively). *Se. perturbans* contained two haplotypes, H63 and H64 from *MGC*^*u*^ and *PHCL*^*a*^, respectively. In the case of *Se. hivernus*, *Se. siamensis*, and *Gr. indica*, each species has a single haplotype, explicitly H61, H62, and H30, originating from *MGC*^*u*^, *TKC*^*a*^, and *MRF*^*f*^, respectively. For *Se.* sp. 1, *PHCL*^*b*^ demonstrated the largest diversity, comprising seven haplotypes (H5, H22, H23, H24, H25, H26, and H27) followed by *PHCS*^*b*^ with four haplotypes (H4, H5, H7, and H8); the clusters *MPC*^*b*^, *PHCL*^*a*^, and *TKC*^*a*^ each contained one haplotype. Meanwhile, *Se.* sp. 2 exhibited only one haplotype (H65) from *PHCL*^*b*^ (Fig. 5A; Table 3).

TCS haplotype network for genus *Phlebotomus* and *Idiophlebotomus* revealed a total of 25 haplotypes categorized into 4 distinct species clusters, including *Phlebotomus barguesae* (4 haplotypes), *Ph. Stantoni* (6 haplotypes), *Idiophlebotomus asperulus* (10 haplotypes), and *Id. longiforceps* (5 haplotypes) (Fig. 5B). For *Ph. barguesae*, the cluster *CUC*^*d*^ contained the largest number of haplotypes, comprising H1, H2, and H3, whereas *PHCS*^*b*^ and *PHCL*^*a*^ included only a single haplotype (H1). For *Ph. stantoni*, *PHCL*^*a*^ and *PHCL*^*b*^ each contained the 2 haplotypes: H16 and H19 in *PHCL*^*a*^, and H17 and H18 in *PHCL*^*b*^, whereas *TGC*^*a*^* PHCS*^*b*^ and *MRF*^*f*^ each contained 1 haplotype, viz. H15, H19, and H20, respectively. In the case of *Id. asperulus*, the cluster *PHCL*^*b*^ housed the majority of haplotypes, totaling eight (H4, H5, H7, H8, H9, H10, H11, and H12), while the cluster *TKC*^*b*^ had just one haplotype (H13). *Id. longiforceps* contained five haplotypes, only from *TKC*^*b*^ (Fig. 5B; Table 3).

### Distribution and percentage of sand fly species identified on the basis of *COI* and *Cytb* sequences

The highest percentages of individuals were recorded for *Phlebotomus barguesae*, which accounted for 38.1% and 33.9% of the total for the *COI* and *Cytb* sequences, respectively. This was followed by *Sergentomyia anodontis*, which accounted for 17.5% and 18.1% of individuals in the *COI* and *Cytb* sequences, respectively. Conversely, the lowest percentages were observed for *Sergentomyia indica*, *Sergentomyia hivernus*, and *Sergentomyia* sp. 2, each contributing 0.5% to the *COI* sequences, and for *Se. indica*, *Se. hivernus*, *Se. siamensis*, and *Sergentomyia* sp. 2, each representing 0.6% of the *Cytb* sequences. The highest number of individuals was found in the *PHCL*^*b*^ cluster for both genes, with 37 individuals for *COI* and 36 for *Cytb*, followed by *PHCL*^*a*^, which contained 26 individuals in each gene. In contrast, the lowest numbers were observed in the *PWHR*^*g*^ cluster, with two individuals for *COI* and one for *Cytb*. Notably, for both genes, *Se. perturbans*, *Se. indica*, *Se. hivernus*, *Sergentomyia* sp. 2, and *Idiophlebotomus longiforceps* were found exclusively in the *MGC*^*c*^, *MRF*^*f*^, *PHCL*^*b*^, and *TKC*^*b*^ clusters, respectively (Table 2).

### Intraspecific and interspecific variation based on *COI* and *Cytb* sequences

Intraspecific variation among phlebotomine sand fly species ranged from 0.1% to 6.8% on the basis of *COI* sequences, and from 0.0% to 5.8% on the basis of *Cytb* sequences. Notably, *Phlebotomus stantoni* exhibited the greatest intraspecific genetic diversity, with genetic divergence of 6.8% for *COI* and 5.8% for *Cytb*, followed by *Sergentomyia barraudi* (3.9%) and *Se. sylvatica* (2.9%) for *COI* and *Se. sylvatica* (3.2%) and *Se. barraudi* (2.8%) for the *Cytb* gene. Generally, intraspecific variation was higher in *COI* compared with *Cytb* for *Ph. barguesae*, *Ph. stantoni*, *Se. barraudi*, and *Se.* sp. 1. Conversely, *Id. asperulus*, *Id. longiforceps*, *Se. anodontis*, and *Se. sylvatica* exhibited lower intraspecific variation in *COI*, while *Se. khawi* and *Se. perturbans* showed identical variation in both genes (Table 4). In relation to interspecific variation, the *COI* gene revealed the highest genetic divergence between *Ph. barguesae* and *Se. barraudi* (23.3%), while the lowest divergence was noted between *Se. hivernus* and *Se.* sp. 1 (3.6%). For the *Cytb* gene, the highest genetic divergence occurred between *Id. longiforceps* and *Se.* *barraudi* (28.9%), with the lowest genetic divergence recorded between *Se. barraudi* and *Se. siamensis* (7.2%). Overall, interspecific variation was generally greater in the *Cytb* gene than in the *COI* gene, unlike intraspecific variation (Table 4).

**Table 4:** Intraspecific and interspecific genetic distances of *COI* (613 bp) and *Cytb* (397–413 bp) sequences among the 12 phlebotomine species in addition to 2 putatively novel species in this study, calculated using the Jukes and Cantor (JC) method

Gene	Species		1		2		3		4		5		6			7		8		9		10		11		12		13	14
*COI*	1.* Gr. indica*		-^a^																										
	2.* Id. asperulus*		0.166 (16.6%)		0.016 (01.6%)^a^																								
		3.* Id. longiforceps*		0.177 (17.7%)		0.148 (14.8%)		0.001 (00.1%)^a,c^																					
		4.* Ph. barguesae*		0.212 (21.2%)		0.225 (22.5%)		0.229 (22.9%)		0.011 (01.1%)^a^																			
		5.* Ph. stantoni*		0.191 (19.1%)		0.210 (21.0%)		0.214 (21.4%)		0.193 (19.3%)		0.068 (06.8%)^a, c^																	
	6.* Se. anodontis*		0.127 (12.7%)		0.168 (16.8%)		0.163 (16.3%)		0.190 (19.0%)		0.193 (19.3%)		0.006 (00.6%)^a^																
	7.* Se. barraudi*		0.137 (13.7%)		0.165 (16.5%)		0.186 (18.6%)		0.233 (23.3%)^b^		0.224 (22.4%)		0.156 (15.6%)			0.039 (03.9%)^a^													
	8.* Se. khawi*		0.156 (15.6%)		0.174 (17.4%)		0.194 (19.4%)		0.198 (19.8%)		0.220 (22.0%)		0.146 (14.6%)			0.158 (15.8%)		0.010 (01.0%)^a^											
	9.* Se. sylvatica*		0.136 (13.6%)		0.190 (19.0%)		0.192 (19.2%)		0.199 (19.9%)		0.198 (19.8%)		0.133 (13.3%)			0.173 (17.3%)		0.178 (17.8%)		0.029 (02.9%)^a^									
	10.* Se. perturbans*		0.143 (14.3%)		0.166 (16.6%)		0.165 (16.5%)		0.203 (20.3%)		0.190 (19.0%)		0.131 (13.1%)			0.155 (15.5%)		0.161 (16.1%)		0.145 (14.5%)		0.008 (00.8%)^a^							
	11. *Se. hivernus*		0.145 (14.5%)		0.167 (16.7%)		0.183 (18.3%)		0.221 (22.1%)		0.198 (19.8%)		0.142 (14.2%)			0.144 (14.4%)		0.139 (13.9%)		0.142 (14.2%)		0.134 (13.4%)		-^a^					
	12.* Se. siamensis*		0.139 (13.9%)		0.174 (17.4%)		0.188 (18.8%)		0.216 (21.6%)		0.204 (20.4%)		0.146 (14.6%)			0.099 (09.9%)		0.160 (16.0%)		0.158 (15.8%)		0.155 (15.5%)		0.137 (13.7%)		-^a^			
	13. *Se.* sp. 1		0.139 (13.9%)		0.163 (16.3%)		0.156 (15.6%)		0.185 (18.5%)		0.195 (19.5%)		0.107 (10.7%)			0.155 (15.5%)		0.168 (16.8%)		0.163 (16.3%)		0.132 (13.2%)		0.036 (03.6%)^b^		0.139 (13.9%)		0.023 (02.3%)^a^	
	14. *Se.* sp. 2		0.139 (13.9%)		0.181 (18.1%)		0.095 (09.5%)		0.214 (21.4%)		0.201 (20.1%)		0.138 (13.8%)			0.170 (17.0%)		0.175 (17.5%)		0.145 (14.5%)		0.123 (12.3%)		0.161 (16.1%)		0.145 (14.5%)		0.125 (12.5%)	-^a^
*Cytb*	1.* Gr. indica*		-^a^																										
	2.* Id. asperulus*		0.242 (24.2%)		0.017 (01.7%)^a^																								
	3.* Id. longiforceps*		0.260 (26.0%)		0.212 (21.2%)		0.002 (00.2%)^a^																						
	4.* Ph. barguesae*		0.208 (20.8%)		0.281 (28.1%)		0.275 (27.5%)		0.000 (00.0%)^a,c^																				
	5.* Ph. stantoni*		0.234 (23.4%)		0.249 (24.9%)		0.271 (27.1%)		0.190 (19.0%)		0.058 (05.8%)^a,c^																		
	6.* Se. anodontis*		0.234 (23.4%)		0.209 (20.9%)		0.233 (23.3%)		0.196 (19.6%)		0.175 (17.5%)		0.007 (00.7%)^a^																
	7.* Se. barraudi*		0.156 (15.6%)		0.251 (25.1%)		0.289 (28.9%)^b^		0.213 (21.3%)		0.208 (20.8%)		0.121 (12.1%)			0.028 (02.8%)^a^													
	8.* Se. khawi*		0.148 (14.8%)		0.218 (21.8%)		0.239 (23.9%)		0.193 (19.3%)		0.199 (19.9%)		0.116 (11.6%)			0.141 (14.1%)		0.010 (01.0%)^a^											
	9.* Se. sylvatica*		0.160 (16.0%)		0.222 (22.2%)		0.233 (23.3%)		0.183 (18.3%)		0.198 (19.8%)		0.128 (12.8%)			0.129 (12.9%)		0.137 (13.7%)		0.032 (03.2%)^a^									
	10.* Se. perturbans*		0.133 (13.3%)		0.216 (21.6%)		0.253 (25.3%)		0.201 (20.1%)		0.197 (19.7%)		0.133 (13.3%)			0.132 (13.2%)		0.108 (10.8%)		0.120 (12.0%)		0.008 (00.8%)^a^							
	11.* Se. hivernus*		0.143 (14.3%)		0.238 (23.8%)		0.273 (27.3%)		0.236 (23.6%)		0.217 (21.7%)		0.134 (13.4%)			0.116 (11.6%)		0.103 (10.3%)		0.156 (15.6%)		0.112 (11.2%)		-^a^					
	12.* Se. siamensis*		0.178 (17.8%)		0.259 (25.9%)		0.283 (28.3%)		0.215 (21.5%)		0.199 (19.9%)		0.111 (11.1%)			0.072 (07.2%)^b^		0.148 (14.8%)		0.128 (12.8%)		0.141 (14.1%)		0.158 (15.8%)		-^a^			
	13. *Se.* sp. 1		0.126 (12.6%)		0.239 (23.9%)		0.252 (25.2%)		0.203 (20.3%)		0.202 (20.2%)		0.143 (14.3%)			0.149 (14.9%)		0.122 (12.2%)		0.157 (15.7%)		0.141 (14.1%)		0.143 (14.3%)		0.153 (15.3%)		0.004 (00.4%)^a^	
	14. *Se.* sp. 2		0.143 (14.3%)		0.238 (23.8%)		0.268 (26.8%)		0.208 (20.8%)		0.211 (21.1%)		0.133 (13.3%)			0.120 (12.0%)		0.122 (12.2%)		0.124 (12.4%)		0.075 (07.5%)		0.121 (12.1%)		0.153 (15.3%)		0.138 (13.8%)	-

Intraspecific distances are highlighted in (a). The highest and lowest interspecific genetic distances are indicated with (b), while the highest and lowest intraspecific genetic distances are highlighted with (c). Intraspecific distances were not analyzed, so only one sequence of each species is indicated with a hyphen (-)

### Polymorphism and genetic diversity analysis

All species under investigation exhibited high haplotype diversity (Hd) for *COI* sequences, with values ranging from 0.492 in *Phlebotomus barguesae* to 1.000 in *Sergentomyia barraudi*. Nucleotide diversity (π) varied from 0.0012 in *Idiophlebotomus longiforceps* to 0.0663 in *Ph. stantoni*. The results of Tajima’s D and Fu and Li’s D* tests for all neutrality tests were not statistically significant (*P* > 0.1). The *COI* analysis revealed negative values for *Ph. barguesae*, *Id. longiforceps*, and *Sergentomyia* sp. 1, suggesting a purifying selection (*P* < 0.01 to *P* < 0.05) in these species. Furthermore, the haplotype diversity for *COI* sequences ranged from 0.464 in *Id. longiforceps* to 1.000 in *Se. barraudi*, except for *Ph. barguesae*, which had a significantly lower haplotype diversity (Hd = 0.085). The nucleotide diversity (π) for this species was found to be 0.0002, while *Ph. stantoni* exhibited the highest nucleotide diversity at 0.0565. The *Cytb* analysis indicated that *Se. anodontis* was the only species to show a negative result (*P* < 0.05). Notably, *Ph. stantoni* displayed a substantially positive result in the Fu and Li’s D* test for *Cytb*, suggesting a genetic tendency toward diversifying selection (Table 5).

**Table 5:** Polymorphism and genetic diversity of eight dominant phlebotomine sand fly species on the basis of *COI* and *Cytb* gene sequences

Species	Gene	Site (bp)	*N*	Diversity indices						Neutrality tests	
				VS	H	Hd (SD)	π (SD)	k		Tajima’s D	Fu and Li’s D*
*Ph. barguesae*	*COI*	613	46	15	3	0.492 (0.047)	0.0018 (0.0009)	1.07246		−2.13293*	−4.72312*
	*Cytb*	398	46	1	2	0.085 (0.055)	0.0002 (0.0001)	0.08502		−0.86025	0.55053
*Ph. stantoni*	*COI*	613	7	86	6	0.952 (0.096)	0.0663 (0.0222)	40.6667		0.48415	1.11276
	*Cytb*	414	7	48	5	0.857 (0.137)	0.0565 (0.0185)	23.0952		0.76122	1.46446*
*Id. asperulus*	*COI*	613	12	28	10	0.970 (0.044)	0.0179 (0.0017)	10.9849		0.82823	0.01805
	*Cytb*	397	12	19	9	0.955 (0.047)	0.0195 (0.0025)	7.72727		0.99861	0.86913
*Id. longiforceps*	*COI*	612	8	2	3	0.679 (0.122)	0.0012 (0.0003)	0.78571		0.06935	−0.14931
	*Cytb*	396	8	2	3	0.464 (0.200)	0.0013 (0.0006)	0.50000		−1.31009	−1.40980
*Se. anodontis*	*COI*	613	29	21	11	0.621 (0.106)	0.0028 (0.0010)	1.70443		−2.40135*	−3.38936*
	*Cytb*	406	29	11	8	0.606 (0.100)	0.0027 (0.0009)	1.11330		−1.95360*	−2.52963*
*Se. barraudi*	*COI*	613	6	71	6	1.000 (0.096)	0.0438 (0.0189)	0.01885		−1.10186	−1.08513
	*Cytb*	412	6	34	6	1.000 (0.096)	0.0327 (0.0130)	13.4667		−0.92837	−0.82956
*Se. khawi*	*COI*	629	13	25	9	0.923 (0.057)	0.0097 (0.0018)	6.07692		−1.06669	−1.31781
	*Cytb*	427	13	10	7	0.731 (0.133)	0.0079 (0.0017)	3.35897		0.16923	0.60929
*Se.* sp. 1	*COI*	624	18	76	13	0.961 (0.030)	0.0187 (0.0097)	11.6536		−2.00179*	−2.87460*
	*Cytb*	410	18	5	5	0.614 (0.117)	0.0020 (0.0006)	0.83660		−1.34363	−1.13794

Statistical significance is shown by numbers in bold and asterisks (*P* < 0.02)

*N*, number of nucleotide sequences analyzed; GC%, percentage of G + C content; VS, number of variable sites; H, number of nucleotide sequence type (ntST); Hd, diversity of nucleotide sequence type; π, nucleotide diversity; k, average number of nucleotide differences; SD, standard deviation. D, Tajima’s and D* Fu and Li’s D*

## Discussion

Phlebotomine sand flies in Thailand are classified into 36 species and five genera: *Phlebotomus*, *Idiophlebotomus*, *Sergentomyia*, *Grassomyia*, and *Chinius* [4, 7, 16, 22]. This study primarily focused on female specimens for both identification and molecular analysis, as male sand flies do not feed on blood and are of lesser medical significance. This approach is consistent with prior research [51, 52], which utilized *COI* and *Cytb* genetic markers. However, the integrative taxonomy approach adopted in this study underscores the importance of male specimens in providing critical morphological information for a comprehensive understanding of local sand fly fauna. Establishing associations between male and female specimens would be invaluable for confirming species identities. Moreover, it is well documented that certain sand fly genera, such as *Trichophoromyia*, *Trichopygomyia*, and *Brumptomyia*, depend significantly on male-specific morphological traits for accurate identification, as males and females in these groups are often isomorphic. Addressing this limitation in the current study highlights the necessity of incorporating male specimens in future research to achieve a more complete and robust taxonomic resolution. The findings of this study revealed four genera—*Sergentomyia*, *Grassomyia*, *Phlebotomus*, and *Idiophlebotomus*—with *Sergentomyia* being the most common, comprising seven species, followed by *Phlebotomus* and *Idiophlebotomus*, each represented by two species. Interestingly, *Id. asperulus* and *Id. longiforceps* were found exclusively in caves, suggesting a habitat restriction for these species related to earlier study [12]. In Thailand, *Sergentomyia* is the most prevalent genus, followed by *Phlebotomus*, while *Chinius* and *Idiophlebotomus* are believed to primarily inhabit caves.

Phylogenetic analyses using *COI* and *Cytb* sequences confirmed the classification of *Se. anodontis*, *Se. khawi*, *Se. perturbans*, *Grassomyia indica*, *Ph. barguesae*, *Id. asperulus*, and *Id. longiforceps*, all with strong bootstrap support (≥ 97%). Additionally, TCS haplotype analysis and species delimitation methods, including ASAP and ABGD, applied across three substitution models (p-distance, JC69, and K2P), further validated these phylogenetic results for both genes. Notably, specimens identified as *Se. hivernus* clustered with *Se. iyengari* sequences from GenBank (LC136905 for *COI* and PQ151883 for *Cytb*), with high bootstrap support (100%). This aligns with findings from Depaquit et al. [53], which suggest that *Se. hivernus* is part of a group that includes sequences labeled as *Se. iyengari* in GenBank, prompting a reassessment of species boundaries. Traditionally, sand fly species have been identified on the basis of distinguishing morphological features such as the pharynx, spermatheca, and cibarium teeth [54, 55]. However, cryptic species complexes and subtle morphological variations often lead to misidentifications. For instance, *Se. gemmea* has frequently been misidentified as *Se. iyengari*, and vice versa. Several studies [22, 56, 57] have suggested that historical records of *Se. iyengari* in Southeast Asia may refer to *Se. khawi*. Additionally, Phumee et al. [18] reported that *Se. iyengari* and *Se. hivernus* were synonymized owing to taxonomic complexities. The findings of this study confirm that *Se. hivernus* has been incorrectly synonymized with *Se. iyengari*, on the basis of both morphological characteristics and molecular data from *COI* and *Cytb* sequences, supported by species delimitation analyses (ASAP and ABGD).

Phylogenetic analysis of *COI* and *Cytb* sequences revealed two well-supported clades of *Se. barraudi*, despite the absence of distinguishable morphological differences. Furthermore, the TCS haplotype network, along with species delimitation methods ASAP and ABGD, corroborated the phylogenetic findings. This raises the possibility of a species complex within *Se. barraudi*, as suggested by a previous study [18]. The genetic diversity observed may indicate the existence of cryptic species, emphasizing the need for further taxonomic research. Species complexes are not uncommon among arthropod vectors, where morphologically identical species may exist within a nominal species, complicating vector incrimination and our understanding of disease transmission [58]. In this study, 11 species were identified using *COI* and 12 using *Cytb*, including two putative novel species (*Se.* sp. 1 and *Se.* sp. 2). The *Cytb* phylogenetic tree effectively distinguished *Se. siamensis* from *Se. barraudi* with high bootstrap support (100%). However, morphological analysis and the *COI* phylogenetic tree failed to differentiate between these two species, tentatively classifying both as *Se. barraudi*. The results, along with previous studies [59], suggest that *Cytb* is a more reliable marker for sand fly species identification. Furthermore, the TCS haplotype network and species delimitation methods (ASAP and ABGD) provided additional support for the separation of *Se. barraudi* and *Se. siamensis* on the basis of both *COI* and *Cytb* genes.

Recent research has highlighted the discovery of several novel sand fly taxa in Thailand through morphological analysis [60], emphasizing the growing importance of molecular techniques in refining species identification. Given that only 37% of nominal sand fly species have been characterized at the molecular level, it is recommended that molecular techniques be integrated with traditional morphological approaches to achieve more accurate identifications, particularly for closely related species [61]. The current study reinforces the necessity of this integrative approach to address taxonomic ambiguities, such as those observed between *Se. barraudi* and *Se. siamensis*. Interestingly, the species delimitation methods (ASAP and ABGD) sorted *Se. sylvatica* into two and three hypothetical species, respectively. Similarly, TCS haplotype network analyses revealed distinct clusters for haplotypes H47, H58, and H60, whereas phylogenetic and morphological analyses supported the existence of a single clade with strong bootstrap values (99.68% for *COI* and 99.78% for *Cytb*). Likewise, phylogenetic analysis of *Ph. stantoni* identified two well-supported clades, despite the absence of obvious morphological differences. Furthermore, TCS haplotype network analyses and both ASAP and ABGD species delimitation methods, based on *COI* and *Cytb* genes, further validated the phylogenetic results. These findings suggest that further taxonomic studies are needed to explore the genetic diversity within these species and resolve remaining taxonomic uncertainties.

In this study, an integrative approach that combined morphological, BLASTN, phylogenetic, and species delimitation analyses confirmed the classification of 11 species across four genera, along with two putative novel species (*Sergentomyia* sp. 1 and *Se.* sp. 2). The distinct diagnostic morphological characteristics of these two novel species confirm that they cannot be assigned to any previously described taxa. However, we recommend conducting formal taxonomic descriptions in future studies to ensure their proper classification. In our study, the thorax, abdomen, and legs were preserved for future molecular analysis. Consequently, the newly identified species may possess diagnostic morphological variations in these structures, which were not examined in the current study and were instead limited to the cibarium, pharynx, and spermatheca. To address this limitation, we recommend the use of nondestructive DNA extraction methods in future investigations. This approach would facilitate the examination of the thorax, abdomen, and legs for potential diagnostic morphological traits, enabling a more comprehensive assessment of species-level variation. To enhance the accuracy of molecular identification, it is recommended to use multiple genetic markers with different evolutionary rates [61–63]. The findings of this study strongly support the adoption of an integrative approach that combines morphological analysis with multilocus genetic data to enhance the accuracy of species identification. Notably, the *COI* phylogenetic tree revealed an unexpected result: haplotype H9 (THSF23-83) was grouped within the *Se. anodontis* clade, while in the *Cytb* phylogenetic tree, it clustered with *Se.* sp. 1. However, the TCS haplotype network analysis, which incorporated both *COI* and *Cytb* markers, indicated that H9 is distinct from both *Se. anodontis* and *Se.* sp. 1. This inconsistency suggests that H9 may represent a unique haplotype or, potentially, an undiscovered species. Further research, including population-level sampling and the use of additional genetic markers, will be necessary to clarify the taxonomic status of this sand fly. The findings of this study also indicate that levels of haplotype diversity (Hd) and nucleotide diversity (π) were generally higher for *COI* sequences compared with *Cytb* sequences, with the exception of two species in the genus *Idiophlebotomus*. On the basis of these results, it is recommended that *COI* be prioritized as a genetic marker for exploring intraspecific variation in phlebotomine sand flies. This recommendation is consistent with the findings of Chen et al. [51], who found that mtDNA *COI* and *Cytb* displayed the highest intraspecific variation in *Phlebotomus chinensis* s.l., further supporting the use of these markers for species identification. However, Depaquit [59] has proposed additional genetic markers that could be informative, though these were not explored in this study. Further research incorporating these markers may provide additional insights into sand fly taxonomy.

## Conclusions

This study presents a comprehensive assessment of phlebotomine sand flies inhabiting caves and wildlife habitats in Thailand by integrating molecular techniques with traditional morphological taxonomy. Through the application of species delimitation methods, including ASAP and ABGD, along with phylogenetic analyses, this study provides strong evidence supporting the existence of cryptic and complex species. Additionally, the identification of two putative novel species, *Sergentomyia* sp. 1 and *Se*. sp. 2, further enhances the understanding of sand fly biodiversity in the region.

## Supplementary Information

**Additional file 1: Supplementary Table S1** Primers for molecular identification of sand flies. **Table S2** Thermal cycling conditions used in this study. **Table S3** Record of reference sequences used in this study

**Additional file 2: Supplementary Table S4** BLASTN and BOLD search results.

**Additional file 3: Supplementary Table S5** GenBank accession no. of deposited sequences obtained from this study.

## Data Availability

The nucleotide sequences obtained in this study were deposited in the GenBank™ database (https://www.ncbi.nlm.nih.gov/nuccore) as provided in Supplementary Table S5. Data are provided within the manuscript or supplementary information files.

## Abbreviations

BLASTN: Basic local alignment search tool for nucleotide
BOLD: Barcode of Life Database
CDC: Center for Diseases Control and Prevention
*COI*: Cytochrome oxidase c subunit I
*Cytb*: Cytochrome b
PCR: Polymerase chain reaction
