# Supplementary material for: An integrative taxonomic approach reveals two putatively novel species of phlebotomine sand fly (Diptera: Psychodidae) in Thailand
Source: Parasit Vectors. 2025 Jan 6;18:1. doi: 10.1186/s13071-024-06640-8 (PMC11702185; doi:10.1186/s13071-024-06640-8)
Supplement: Supplementary file 1 — Additional file 1: Supplementary Table S1 Primers for molecular identification of sand flies. Table S2 Thermal cycling conditions used in this study. Table S3 Record of reference sequences used in this study [file 13071_2024_6640_MOESM1_ESM.docx]

**Table S1** Primers for molecular identification of sand flies

| Target gene (mtDNA) | Primer name | Sequence (5’-3’) | bp | Tm (°C) | Product size (bp) | Reference |
| --- | --- | --- | --- | --- | --- | --- |
| COI | LepF： | 5’-ATTCAACCAATCATAAAGATATTGG-3’ | 25 | 48.8 | 650 | (Depaquit et al., 2009) |
|  | LepR： | 5’- AAACTTCTGATGTCCAAAAAATCA-3’ | 26 | 50.7 |  |  |
| Cytb | N1N-PDR: | 5’-CAYATTCAACCWGAATGATA-3’ | 20 | 45.9 | 500 | (Esseghir et al., 1997) |
|  | C3B-PDR: | 5’-GGTAYWTTGCCTCGAWTTCGWTATGA-3’ | 26 | 56.6 |  |  |

**Table S2** Thermal cycling conditions used in this study

|  |  | *COI* gene | | |  | *Cytb* gene | | |
| --- | --- | --- | --- | --- | --- | --- | --- | --- |
| Steps |  | Temp (°C) | Time (min) | Tmp/Cycl |  | Temp (°C) | Time (min) | Tmp/Cycl |
| Initial Denaturation |  | 94.0 °C | 2:00 | 1TmP |  | 94.0 °C | 2:00 | 1TmP |
| Denaturation |  | 98.0 °C | 0:10 | 3TmP 35 Cyc |  | 98.0 °C | 0:10 | 3TmP 35 Cyc |
| Annealing |  | 50.0 °C | 0:30 |  |  | 50.0 °C | 0:30 |  |
| Extension |  | 68.0 °C | 1:00 |  |  | 68.0 °C | 1:00 |  |
| Final Extension |  | 68.0 °C | 7:00 | 2TmP 1 Cyc |  | 68.0 °C | 7:00 | 2TmP 1Cyc |
|  |  | 12.0°C | Infinite time |  |  | 12.0°C | Infinite time |  |

**Table S3** Record of reference sequences used in this study

| ***Cytb*** | | | | ***COI*** | | | |
| --- | --- | --- | --- | --- | --- | --- | --- |
| *Sergentomyia anodontis* | MN853016 | 266 | Thailand | *Sergentomyia anodontis* | MK807983 | 708 | Thailand |
| *Sergentomyia anodontis* | OQ784731 | 318 | Thailand | *Sergentomyia sylvatica* | OP879807 | 563 | Thailand |
| *Sergentomyia anodontis* | OQ784726 | 318 | Thailand | *Sergentomyia sylvatica* | [BOLD:ACZ0089](http://boldsystems.org/index.php/Public_BarcodeCluster?clusteruri=BOLD:ACZ0089) | 607 | Thailand |
| *Sergentomyia anodontis* | OQ784694 | 318 | Thailand | *Sergentomyia perturbans* | LC136897 | 650 | Thailand |
| *Sergentomyia khawi* | MK548554 | 432 | Thailand | *Sergentomyia barraudi* | LC136903 | 650 | Thailand |
| *Sergentomyia khawi* | MK548553 | 432 | Thailand | *Sergentomyia indica* | LC136896 | 650 | Thailand |
| *Sergentomyia khawi* | MN853003 | 492 | Thailand | *Sergentomyia iyengari* | LC136905 | 650 | Thailand |
| *Sergentomyia barraudi* | MG770903 | 414 | Thailand | *Sergentomyia hivernus* | OP879776 | 708 | Thailand |
| *Sergentomyia barraudi* | MK442494 | 488 | Thailand | *Sergentomyia hivernus* | MN850820 | 549 | Thailand |
| *Sergentomyia siamensis* | OQ784738 | 318 | Thailand | *Sergentomyia khawi* | OP879772 | 573 | Thailand |
| *Sergentomyia sclerosiphon* | MK465143 | 430 | Madagascar | *Sergentomyia khawi* | MN850819 | 549 | Thailand |
| *Sergentomyia hivernus* | OP375564 | 534 | Thailand | *Phlebotomus barguesae* | FJ348734 | 688 | Thailand |
| *Sergentomyia hivernus* | MK548571 | 429 | Thailand | *Phlebotomus stantoni* | LC136899 | 650 | Thailand |
| *Sergentomyia hivernus* | MG770916 | 372 | Thailand | *Phlebotomus stantoni* | LC136898 | 650 | Thailand |
| *Grassomyia sp.* | OQ784723 | 318 | Thailand | *Phlebotomus stantoni* | MF966705 | 631 | China |
| *Sergentomyia indica* | MK460566 | 479 | Thailand | *Idiophlebotomus asperulus* | MK807979 | 708 | Thailand |
| *Sergentomyia indica* | OK398069 | 490 | Thailand | *Idiophlebotomus longiforceps* | MN850817 | 549 | Thailand |
| *Phlebotomus barguesae* | MN853023 | 266 | Thailand | *Idiophlebotomus longiforceps* | [BOLD:AEG3626](http://boldsystems.org/index.php/Public_BarcodeCluster?clusteruri=BOLD:AEG3626) | 549 | Thailand |
| *Phlebotomus barguesae* | KM409509 | 418 | Thailand | *Lutzomyia longipalpis* (Outgroup) | JQ769143 | 711 | USA |
| *Phlebotomus stantoni* | MG770905 | 415 | Thailand |  |  |  |  |
| *Phlebotomus stantoni* | MK431379 | 486 | Thailand |  |  |  |  |
| *Phlebotomus stantoni* | MG770904 | 412 | Thailand |  |  |  |  |
| *Idiophlebotomus asperulus* | MN853020 | 266 | Thailand |  |  |  |  |
| *Idiophlebotomus longiforceps* | MN853019 | 266 | Thailand |  |  |  |  |
| *Lutzomyia gomezi*(Outgroup) | EF012222 | 479 | Colombia |  |  |  |  |
